# Intelligent De Novo Design of Novel Antimicrobial Peptides against Antibiotic-Resistant Bacteria Strains

**DOI:** 10.3390/ijms24076788

**Published:** 2023-04-05

**Authors:** Tzu-Tang Lin, Li-Yen Yang, Chung-Yen Lin, Ching-Tien Wang, Chia-Wen Lai, Chi-Fong Ko, Yang-Hsin Shih, Shu-Hwa Chen

**Affiliations:** 1 Institute of Information Science, Academia Sinica, Taipei 11529, Taiwan; 2 Department of Agricultural Chemistry, National Taiwan University, Taipei 10617, Taiwan; 3 TMU Research Center of Cancer Translational Medicine, Taipei Medical University, Taipei 110301, Taiwan

**Keywords:** antimicrobial peptides, generative adversarial network, drug design

## Abstract

Because of the growing number of clinical antibiotic resistance cases in recent years, novel antimicrobial peptides (AMPs) may be ideal for next-generation antibiotics. This study trained a Wasserstein generative adversarial network with gradient penalty (WGAN-GP) based on known AMPs to generate novel AMP candidates. The quality of the GAN-designed peptides was evaluated in silico, and eight of them, named GAN-pep 1–8, were selected by an AMP Artificial Intelligence (AI) classifier and synthesized for further experiments. Disc diffusion testing and minimum inhibitory concentration (MIC) determinations were used to identify the antibacterial effects of the synthesized GAN-designed peptides. Seven of the eight synthesized GAN-designed peptides displayed antibacterial activity. Additionally, GAN-pep 3 and GAN-pep 8 presented a broad spectrum of antibacterial effects and were effective against antibiotic-resistant bacteria strains, such as methicillin-resistant *Staphylococcus aureus* and carbapenem-resistant *Pseudomonas aeruginosa*. GAN-pep 3, the most promising GAN-designed peptide candidate, had low MICs against all the tested bacteria. In brief, our approach shows an efficient way to discover AMPs effective against general and antibiotic-resistant bacteria strains. In addition, such a strategy also allows other novel functional peptides to be quickly designed, identified, and synthesized for validation on the wet bench.

## 1. Introduction

Over the past decades, the increasing number of clinical antibiotic resistance cases has driven the demand for novel antibiotic discoveries and applications [1,2]. Antimicrobial peptides (AMPs) are natural peptides that are less likely to cause drug resistance in bacteria [3,4]. However, discovering new AMPs through the traditional approach of collecting peptides from various organisms is usually time consuming and costly. Therefore, a deep learning model approach was proposed in this study for in silico AMP design to accelerate the AMP discovery process.

Artificial intelligence technologies and machine learning applications (AI/ML) are fundamentally revolutionizing the drug development process [5,6]. For example, Zeng et al., used a knowledge graph embedding model to prioritize potential candidates to develop a COVID-19 therapy [7], and Jiang et al., utilized a convolutional graph network to predict the effectiveness of synergistic drug combinations against cancers [8]. AI/ML can also be applied to predict various biological and chemical properties, such as protein structure, molecular function, aqueous solubility, and minimum inhibitory concentration (MIC) [9,10,11,12], and can be used to construct biomolecule classifiers to identify protein families, the surfaceome, protein–protein interactions, human leukocyte antigen complexes, and AMPs [13,14,15,16,17,18,19,20,21,22]. The MIC is the lowest concentration of a chemical that prevents visible growth of bacteria. MICs are usually the starting point for larger preclinical evaluations of novel antimicrobial agents [23], and are used to ensure that antibiotics are chosen efficiently to increase the success of treatment.

Several researchers have adopted in silico methods to support and accelerate AMP candidates’ discovery. Some methods were based on computational algorithms [24,25], and many studies have utilized DNNs to generate peptides for AMP design. For example, Müller et al., trained a generative long short-term memory model to capture patterns of AMPs [26], and Dean and Walper utilized a variational autoencoder (VAE) to generate a latent space to create new AMPs from known AMPs [27]. A deep neural network (DNN) named AI4AMP was recently proposed for predicting AMP activity [28]. Van Oort et al., presented AMPGAN, a bidirectional conditional generative adversarial network (BiCGAN)-based approach for rational AMP design. This model uses generator–discriminator dynamics to learn data-driven priors and controls, generated using conditioning variables with in silico validation only [29]. Recently, a generative adversarial network (GAN), a neural network architecture composed of a generative and discriminative model, was used for DNA and protein design [30,31,32,33,34,35,36]. The deep convolutional GAN (DCGAN) was applied in various image generation tasks [37].

This research generates novel AMP candidates based on a deep convolutional generative adversarial network (DCGAN) with known AMPs. Here, we introduced a Wasserstein generative adversarial network with gradient penalty (WGAN-GP) [38], an improved GAN performing stability and reducing mode collapse problems in the original GAN architecture. The collected AMPs were first encoded by PC6, a protein-encoding method proposed in a previous study, which entails the transformation of each peptide into a matrix to consider the order of amino acids and the physicochemical properties of each amino acid. Then, a WGAN-GP-based model was proposed to generate AMPs. After training the model with these encoded AMPs, the generator could identify AMP candidates with random noise as its input. Then, these peptides were predicted by AI4AMP [39] to evaluate the activity of the generated AMP candidates in silico before wet-bench experiments. Eight GAN-designed peptides, named GAN-pep 1–8, and a known AMP with high antimicrobial activity (polyphemusin I) were used to test their activities on *Escherichia coli*, methicillin-susceptible and -resistant strains of *Staphylococcus aureus*, and carbapenem-susceptible and -resistant strains of *Pseudomonas aeruginosa* by using the disc diffusion method and MIC determination.

## 2. Results and Discussion

### 2.1. Evaluating GAN-Designed Peptides In Silico

**Figure 1:**
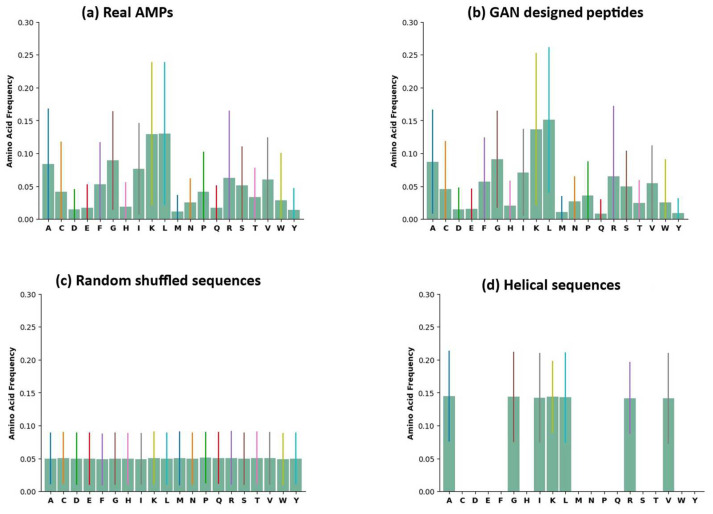
Distribution of amino acid frequencies in (**a**) real AMPs, (**b**) GAN-designed peptides, (**c**) random shuffled sequences, and (**d**) helical peptides. The bar of standard deviation for each amino acid was labeled in different colors for easy comparison among these 4 distributions.

**Figure 2:**
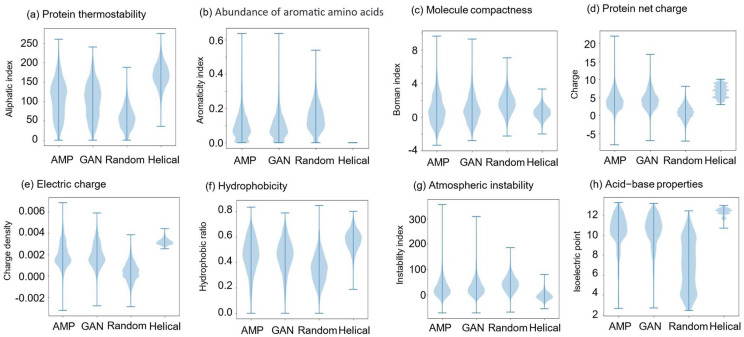
Violin plots of physicochemical properties in four peptide groups. AMP: real AMPs; GAN: GAN-designed peptides; Random: randomly shuffled sequences; Helical: helical sequences. (**a**) Protein thermostability; (**b**) Abundance of aromatic amino acids; (**c**) Molecule compactness; (**d**) Protein net charge; (**e**) Electric charge; (**f**) Hydrophobicity; (**g**) Atmospheric instability; (**h**) Acid-base properties.

**Figure 3:**
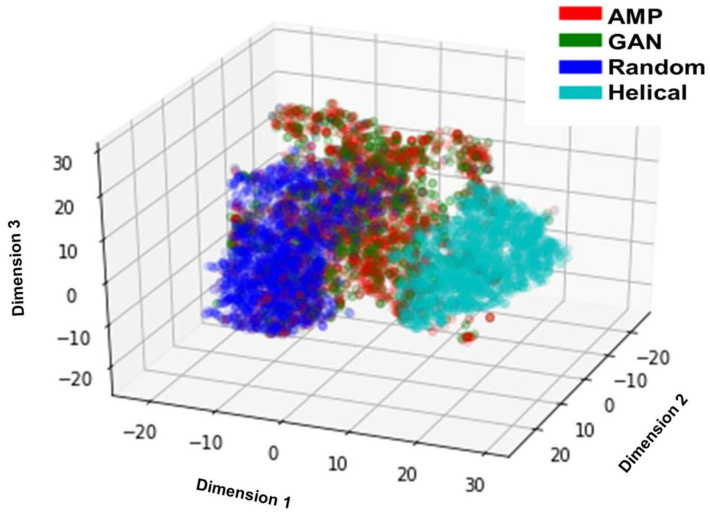
t-SNE plot of four peptide groups. AMP: real AMPs; GAN: GAN-designed peptides; Random: randomly shuffled sequences; Helical: helical sequences.

The amino acid distribution of four groups of peptides is presented in Figure 1, demonstrating that the amino acid composition of the real AMPs and GAN-designed peptides had an extremely similar pattern compared with randomly shuffled and helical sequences. It suggests that the GAN model can capture the pattern in sequence composition. This result indicates that the model neither generates random sequences nor learns the patterns for the alpha helix. The violin plots of eight physicochemical properties of the four groups of peptides are illustrated in Figure 2. The eight physicochemical properties used to evaluate the peptides were the aliphatic index, aromaticity, Boman index, charge density, charge, hydrophobic ratio, instability index, and isoelectric point [40]. The distribution pattern of the GAN-designed peptides resembled that of the real AMPs. This pattern suggests that the GAN model can produce peptides with physicochemical properties crucial for antimicrobial activity. Then, the eight physicochemical features were reduced to three dimensions through t-distributed stochastic neighbor embedding (t-SNE) and were visualized using Matplotlib [41,42]. The t-SNE plot of the four groups of peptides is presented in Figure 3. The real AMPs and the GAN-designed peptides are closely clustered and distinct from the two groups in the embedded space, demonstrating that the GAN-designed peptides possessed similar properties to the real AMPs.

### 2.2. Evaluating GAN-Designed Peptides In Vitro

Various concentrations (7.8125 to 500 μg/mL) of GAN-designed peptides (GAN-pep 1–8) were prepared for a disc diffusion assay, in which a known AMP (polyphemusin I) was used as the positive control and bovine serum albumin was used as the negative control. The results of the disc diffusion susceptibility test of the GAN-designed peptides, the positive control peptide, and negative control peptide at various concentrations against several bacteria, namely, *E. coli*, the clinical isolates of methicillin-susceptible *S. aureus*, methicillin-resistant *S. aureus*, carbapenem-susceptible *P. aeruginosa*, and carbapenem-resistant *P. aeruginosa*, are presented in Supplementary Figures S1–S6. As presented in Supplementary Figure S1, at least one concentration of polyphemusin I and GAN-pep 2, 3, 4, 5, 7, and 8 inhibited the tested Gram-negative bacterium *E. coli*. As presented in Supplementary Figure S2, at least one concentration of polyphemusin I GAN-pep 3, 4, 6, and 8 inhibited the tested Gram-positive bacterium methicillin-susceptible *S. aureus*. As presented in Supplementary Figure S3, at least one concentration of polyphemusin I and GAN-pep 3, 6, and 8 inhibited the Gram-positive bacterium methicillin-resistant *S. aureus*. As presented in Supplementary Figure S4, only GAN-pep 2, 3, 4, and 8 inhibited the tested Gram-negative bacterium carbapenem-susceptible P. aeruginosa at one or more concentrations. As presented in Supplementary Figure S5, only GAN-pep 2, 3, and 8 inhibited the tested Gram-negative bacterium carbapenem-resistant *P. aeruginosa* at one or more concentrations. Overall, GAN-pep 3 and GAN-pep 8 had the broadest antibacterial effects against all tested bacteria. According to the sequence alignment, the most similar to GAN-pep 3 was a Cecropin A-melittin hybrid protein (Accession: ABB29918.1) with only around 60% identities. This hybrid protein is a synthetic construct and is expressed in *Pichia pastoris*. Cecropin is an antimicrobial peptide with a secondary structure that includes two α helices from the hemolymph of *Hyalophora cecropia* (a kind of moth). It also shows anticancer activity. Moreover, melittin is also a natural peptide from bee venom which induces apoptosis and shows antibacterial activity against the strain of Staphylococcus aureus (strain 80) resistant to penicillin [43]. For the GAN-pep 8, nothing similar was identified by BLASTP on the nr database with E < 1.

The MIC of each peptide for each microorganism used in this study is presented in Table 1. Polyphemusin I and GAN-pep 1, 2, 3, 4, 5, 7, and 8 had MICs ranging from 0.7 to 22.5 μg/mL against the Gram-negative bacterium *E. coli*. Polyphemusin I exhibited excellent antibacterial activity against *E. coli*, with a 0.7 μg/mL MIC. GAN-pep 3 and 8 had MICs ranging from 6 to 15 μg/mL against the tested Gram-positive bacterium methicillin-susceptible *S. aureus,* and had an MIC of 45 μg/mL against the tested Gram-positive bacterium methicillin-resistant *S. aureus*. GAN-pep 2, 3, and 4 had MICs ranging from 3 to 50 μg/mL against the tested Gram-negative bacterium carbapenem-susceptible *P. aeruginosa,* and MICs ranging from 3 to 35 μg/mL against the tested Gram-negative bacterium carbapenem-resistant *P. aeruginosa*.

Seven out of the eight GAN-designed peptides exhibited antimicrobial activity against at least one strain of bacteria. This strategy demonstrates that the GAN model can successfully design novel sequence patterns with antimicrobial activity. GAN-pep 3 and GAN-pep 8 displayed broad and practical antibacterial activities, had inhibitory effects against both Gram-negative and Gram-positive bacteria, and inhibited bacteria strains that had developed antibiotic resistance.

## 3. Materials and Methods

### 3.1. Collecting AMPs to Train the Model

The antibacterial AMPs from four AMP databases were collected [44,45,46,47]. Sequences with lengths shorter than ten or with uncommon amino acids, such as B, J, O, U, Z, or X, were excluded. Given the difficulty and cost of synthesizing long peptides, only AMPs shorter than 30 amino acids were selected; finally, 3195 AMPs were selected for this study.

### 3.2. The Architecture of the Proposed GAN

The fundamental idea behind a GAN involves a discriminator and a generator [30]. In this study, the discriminator is trained to learn features from real data, namely, the collected AMPs. The generator is designed to produce fake data in order to deceive the discriminator, viz., to create data that resemble real AMPs. Interactions between the discriminator and the generator are expected to improve the performance of both models. Mathematically, the discriminator is updated with each epoch to maximize the discriminator score of the real data and minimize the score of the fake data. In the meantime, the generator is updated to maximize the discriminator score, as well. The proposed GAN model for generating AMPs was based on the DCGAN, a convolutional network-based GAN [37]. The kernel size, stride, and padding parameters in transposed convolution layers were adjusted to fit the data size. The method proposed in WGAN-GP was used to avoid mode collapse [38]. The following equation shows the loss function of traditional GAN (Equation (1)):

(1) $$min_{G}max_{D}\left\{E_{x\sim {\mathbb{P}}_{r}}\left[\log\left(D\left(x\right)\right)\right]+E_{\tilde{x}\sim {\mathbb{P}}_{g}}\left[\log\left(1-D\left(\tilde{x}\right)\right)\right]\right\}$$

GAN is the min–max game between the generator (*G*) and the discriminator (*D*), where ${\mathbb{P}}_{r}$ represents the training data distribution, ${\mathbb{P}}_{g}$ represent generated data distribution, x represents real data sampled from ${\mathbb{P}}_{r}$, $\tilde{x}$ represents generated data sampled from ${\mathbb{P}}_{g}$, and E is the expectation operator. The generator produces fake data with noise to increase the diversity of fake data. As described previously, the discriminator is trained to maximize the probability of identifying and generating training data correctly, and the generator is trained to minimize $\log\left(1-D\left(\tilde{x}\right)\right)$ simultaneously to make training data and generated data more similar [30]. To avoid problems such as mode collapse during model training, the loss function of WGAN has been proposed [48] based on the Kantorovich–Rubinstein duality to the following (Equation (2)):

(2) $$min_{G}max_{{\left\|D\right\|}_{L}\leq 1}\left\{E_{x\sim {\mathbb{P}}_{r}}\left[D\left(x\right)\right]-E_{\tilde{x}\sim {\mathbb{P}}_{g}}\left[D\left(\tilde{x}\right)\right]\right\}$$

where *D* is a set of 1-Lipschitz functions to define two data distribution distances better using Earth–Mover distance. To further solve undesired behaviors, such as gradient vanishing and training instability, WGAN-GP [38] has been proposed, which adopts an alternative weight clipping gradient penalty (Equation (3)):

(3) $$L=E_{\tilde{x}\sim {\mathbb{P}}_{g}}\left[D\left(\tilde{x}\right)\right]-E_{x\sim {\mathbb{P}}_{r}}\left[D\left(x\right)\right]+\lambda E_{\tilde{x}\sim {\mathbb{P}}_{\tilde{x}}}[{\left(\|\nabla_{\tilde{x}}D{(\tilde{x})\|}_{2}-1\right)}^{2}]$$

where ${\mathbb{P}}_{\tilde{x}}$ is sampled uniformly between ${\mathbb{P}}_{r}$ and ${\mathbb{P}}_{g}$ distributions, λ is a penalty coefficient, and a gradient penalty of $\lambda E_{\tilde{x}\sim {\mathbb{P}}_{\tilde{x}}}[{\left(\|\nabla_{\tilde{x}}D{(\tilde{x})\|}_{2}-1\right)}^{2}]$ in WGAN achieves Lipschitz continuity. In addition, unlike other GANs using batch normalization to help model stabilizing during training, WGAN-GP instead uses layer normalization to fit the gradient penalty, processing each input independently. Hence, we adopted WGAN-GP for better-performing stability and to reduce mode collapse problems.

**Figure 4:**
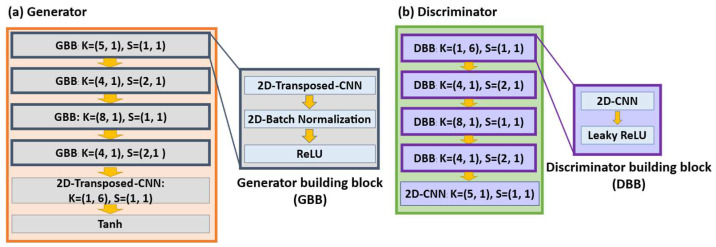
The proposed architecture of generator and discriminator. (**a**) Generator; (**b**) Discriminator.

The proposed generator consisted of five transposed convolution blocks. The first four building blocks comprised a two-dimensional (2D) transposed convolution layer, a 2D batch normalization layer, and an activation layer called the rectifier linear unit (ReLU). The last two blocks were a 2D transposed convolution layer and a tanh activation layer, respectively. Five convolution blocks formed the proposed discriminator, which included the first four building blocks, comprising a 2D convolutional layer and a leaky ReLU, and the last block of a 2D convolutional layer. The training data were first converted into vectors with shapes of (1, 30, 6), denoted by the real PC6 matrix. The generator took a noise vector with a shape of (100, 1, 1) and mapped it to a vector with (1, 30, 6), denoted by the false PC6 matrix. The discriminator took in either the real PC6 matrix or the false PC6 matrix and converted it into a vector with a shape of (1, 1, 1), representing the discriminator’s data score. The proposed architectures of the generator and discriminator are presented in Figure 4, where *K* indicates the kernel size and *S* indicates the stride value.

### 3.3. Mechanism of AMP Production

**Figure 5:**
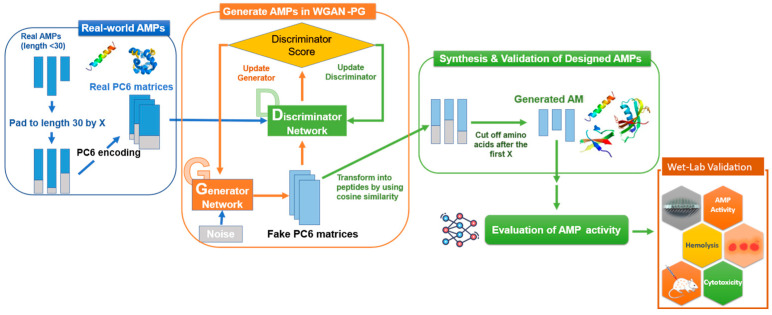
Workflow of training WGAN-PG to generate AMPs with filters of assessment on AMP activity in silico.

For transforming peptides into numeric matrices, the PC6 protein-encoding method in our previous study was used to encode the peptides [39]. This PC6 protein-encoding method transformed a peptide of length *k* into a (6, *k*) shape matrix to store the physicochemical properties of the peptide according to the amino acid sequence. Six physicochemical property values in the PC6 table were scaled to a range of −1 to 1 to ensure every property had a balanced numerical effect in model training and to fit the tanh activation function in the last layer of the generator. Sequences shorter than 30 were padded with a zero-vector “X” at the end to make a sequence length of 30. Each AMP was then transformed into a real PC6 matrix with a shape of (1, 30, 6) using the scaled PC6 table. Then, this matrix was fed into the discriminator and produced discriminator scores. The false PC6 matrices were fed into the discriminator and produced discriminator scores. The cosine similarity converted the generated peptides from the false PC6 matrices. Each row’s six generated physicochemical values were converted into an amino acid with the highest cosine similarity. If the six generated physicochemical values were like a zero vector, the corresponding residue site would be converted into “X”. After the first “X” and including itself, any amino acid would be discarded. Figure 5 presents the overall workflow of training the GAN to generate AMPs.

### 3.4. Training Process

**Figure 6:**
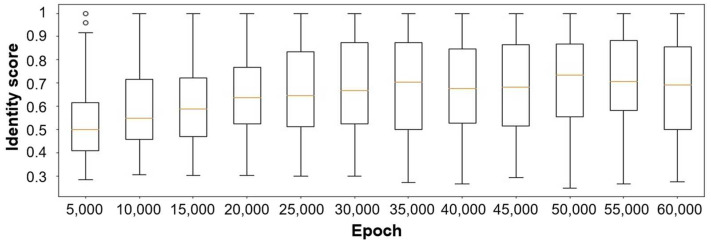
Boxplot of maximum identity score distribution of generated peptides and real AMPs throughout the training process. Outliers are indicated as “o”.

Following WGAN-GP, the generator and discriminator’s training steps were set to 1:5 [38], and the batch size was 128. The Adam algorithm was applied as the optimizer for both models, with the learning rate as 1 × 10^−4^, β_1_ as 0, and β_2_ as 0.9 [49]. Every 5000 epochs, the 128 generator-designed sequences were evaluated. A fixed noise vector was used as the input for these generators, and the outputs were transformed into peptides. The identity between the generated peptide and the real AMP was then evaluated by comparing the ratio of the same amino acid on the overlapped section. Each generated peptide was compared with every AMP in the dataset, producing 3195 identity scores. The identity score for the generated AMP was defined as its maximum identity scored within the real AMP dataset. The training process consisted of 60,000 epochs. As presented in Figure 6, the identity score of the 128 test sequences produced by the current generators improved with increased training steps, and it stabilized after approximately 50,000 training epochs.

### 3.5. Evaluation of GAN-Designed Sequences

The peptide properties of the GAN-designed peptides, real AMPs, randomly shuffled sequences, and helical sequences were compared with the real AMPs to evaluate whether the proposed GAN model had learned to generate peptides that had similar properties to actual AMPs. The randomly shuffled sequences were randomly generated peptides with equal probabilities of all residues to ensure that the proposed model did not merely generate random sequences. Because many AMPs folded into alpha helices [50], the model may have only learned the patterns of helices rather than the patterns having antimicrobial properties. The peptides were compared with helical sequences generated by placing lysine or arginine on every three or four amino acids. Randomly shuffled and helical sequences were generated in “sequences.random” and “sequences.helix” modules with 10 to 30 amino acid lengths using the modlAMP package [51]. A total of 3195 randomly shuffled sequences, 3195 helical sequences, and 3195 GAN-designed peptides were generated to compare with 3195 real AMPs. Each sequence was converted into a data matrix in PC6 encoding [28], namely, a data frame to carry six selected physicochemical properties of the corresponding amino acids. Then, we calculated the cosine similarity in Python using the cosine_similarity function from the sklearn.metrics.pairwise module as an identity score for each peptide pair based on their physiochemical properties for GAN-designed and real peptides.

### 3.6. GAN-Designed Sequence Selection for Experimental Validation

After removing duplicated peptides from the 3195 GAN-designed peptides, 1970 GAN-designed peptides remained. Eight were selected according to the following criteria to ascertain whether the produced sequences had antimicrobial activities. The GAN-designed peptides were kept only if eight physicochemical properties, namely, charge, charge density, isoelectric point, instability index, aromaticity, aliphatic index, Boman index, and hydrophobic ratio [40], were within the range of the mean value plus or minus one standard deviation of those of the real antimicrobial peptides. These physicochemical properties were calculated using the modlAMP package V4.3.0 [51]. Subsequently, the remaining produced sequences were fed into AI4AMP [39], a CNN model for predicting the probability of a peptide with antimicrobial activity. The GAN-designed peptide was selected if the probability of having antimicrobial activity was greater than 0.98. The 1970 GAN-designed peptides were classified into three categories according to their identity scores. Very similar sequences were those with identity scores ranging from 80% to 98%, moderately similar sequences had identity scores from 40% to 60%, and dissimilar sequences had scores lower than 20%. To determine whether sequences that were unlike the real AMPs nonetheless possessed antibacterial properties, twenty-one sequences from the very similar sequence category were selected. In addition, 13 sequences from the moderately similar sequence category were also selected. No sequences were selected from the dissimilar sequence category. Four sequences from the very similar sequence category (GAN-pep 1–4) and four sequences from the moderately similar sequence category (GAN-pep 5–8) were then selected for synthesis for further antimicrobial experiments.

### 3.7. Strains and Reagents

The bacterial strains used for the antimicrobial activity assays were *E. coli* (SG13009), the clinical isolates of methicillin-susceptible *S. aureus* (S01-10-0202), methicillin-resistant *S. aureus* (N07-10-0043), carbapenem-susceptible *P. aeruginosa* (S07-10-0059), and carbapenem-resistant *P. aeruginosa* (M06-06-0213), which were obtained from Dr. Ying-Lien Chen at National Taiwan University. All strains were grown aerobically on an orbital shaker (150 rpm) at 37 °C in Luria–Bertani (L.B.) broth (BD Difco, Franklin Lakes, NJ, USA) overnight. Antibacterial assays were performed to determine the antimicrobial activity of the GAN-designed peptides (GAN-pep 1–8) and the control peptide (Polyphemusin I, UniProt Accession: P14215). Experiments were conducted in at least triplicate in order to obtain consistent judgements for these qualitative assays.

### 3.8. Antimicrobial Assays

The GAN-designed peptides’ antibacterial potential was evaluated using a disc diffusion assay. The bacteria were grown in L.B. broth at 37 °C with agitation. The strain growth was measured turbidimetrically at OD_600_, and at least three separate experiments were conducted for each test organism. Nutrient agar was prepared by mixing agar, sodium chloride, yeast extract, and peptone in distilled water (pH 7.2). Subsequently, a bacterial suspension (100 μL, 1 × 10^8^ CFU/mL) was added and spread on the L.B. agar. Sterilized filter discs (with diameters of 6 mm) were then placed on the agar surface filled with 40 μL of peptide samples. The Petri dish was incubated overnight at 37 °C to observe the inhibitory area.

MIC assays were conducted to determine the antibacterial spectrum of these peptides. MIC is defined as the minimum concentration of a reagent, viz., peptide, in this study, required to inhibit bacterial growth after overnight incubation. Microbial strains were cultured in an L.B. medium, and midlogarithmic-phase organisms were used in the antibacterial assays. All bacteria were inoculated in an L.B. medium (approximately 10^5^ CFU/mL), and MIC assays were performed with various concentrations of each peptide. All activity measurements were conducted at least three times.

## 4. Conclusions and Future Work

This study proposed a new AMPs design method to support AMP discovery in an AI-guided approach. The antibacterial AMPs were encoded through the PC6 protein-encoding method and were then used to train the proposed GAN model using a modified DCGAN architecture based on WGAN-GP [37,38]. The trained generator produced the AMP candidates, which were evaluated by comparing the peptide amino acid distribution and physicochemical properties of four peptide groups. Additionally, a deep learning model named AI4AMP was used to predict the AMP activity of the GAN-designed peptides [39]. The eight GAN-designed peptides (GAN-pep 1–8) predicted to have antimicrobial activities with probabilities greater than 0.98 were synthesized. Finally, the AMP activities of GAN-pep 1–8 were examined using disc diffusion testing and MIC determination. Seven of the eight synthesized GAN-designed peptides exhibited antibacterial activities, demonstrating that the proposed GAN model could design AMPs with antibacterial effects. Among them, GAN-pep 3 and GAN-pep 8 possessed a broad spectrum of antibacterial effects and were effective against antibiotic-resistant bacterial strains, such as methicillin-resistant *S. aureus* and carbapenem-resistant *P. aeruginosa*. GAN-pep 3, the most promising AMP candidate, had lower MICs against *S. aureus* and *P. aeruginosa* than the positive control AMP. 

For transforming GAN-designed peptides into potential drugs, more prediction models/classifiers (hemolysis, sensitivity to Gram+/−, MIC for specific species, etc.) and additional experiments are required to speed up the whole process of screening. For example, hemolysis is a significant factor that causes safety concerns and hinders AMPs from passing later phases of drug development. Experiments on the hemolysis effect of those GAN-designed peptides should be executed. The proposed approach could generate many short peptides and may be used to design and identify peptides with antiviral, antifungal, and anticancer effects, and even various therapeutic applications.

## Figures and Tables

**Table 1:** Antibacterial activity (MIC, μg/mL) of GAN-designed peptides and one known antibiotic peptide from horseshoe crab (polyphemusin I) on five tested bacteria species/strains.

Bacteria Species	*E. coli*	*S. aureus*		*P. aeruginosa*	
Strain	SG13009	MSSA S01-10-0202 ^※^	MRSA N07-10-0043 ^※※^	S07-10-0059 *	M06-06-0213 **
**polyphemusin I**	0.7	>50	>50	>50	>50
**GAN-pep 1**	>50	>50	>50	>50	>50
**GAN-pep 2**	2	>50	>50	50	5
**GAN-pep 3**	2	6	45	3	3
**GAN-pep 4**	2	>50	>50	50	35
**GAN-pep 5**	22.5	>50	>50	>50	>50
**GAN-pep 6**	>50	>50	>50	>50	>50
**GAN-pep 7**	>50	>50	>50	>50	>50
**GAN-pep 8**	15	15	45	>50	>50

***Remarks* ^※^**: *S. aureus* N07-10-0202 is a methicillin-susceptible strain which is also known as MSSA. **^※※^**: *S. aureus* N07-10-0043 is a methicillin-resistant strain which is also known as MRSA. *: *P. aeruginosa* M06-10-0059 is a carbapenem-susceptible strain. **: *P. aeruginosa* M06-06-0213 is a carbapenem-resistant strain.

## Data Availability

The code for training the GAN model for AMP design is publicly available at https://github.com/lsbnb/amp_gan (accessed on 15 February 2023). Video clips of this study can be found at https://www.youtube.com/watch?v=ADn36C1pbHs (accessed on 15 February 2023).

## Supplementary Materials

The following supporting information can be downloaded at: https://www.mdpi.com/article/10.3390/ijms24076788/s1.
