# Supplementary material for: Intelligent De Novo Design of Novel Antimicrobial Peptides against Antibiotic-Resistant Bacteria Strains
Source: Int J Mol Sci. 2023 Apr 5;24(7):6788. doi: 10.3390/ijms24076788 (PMC10095442; doi:10.3390/ijms24076788)
Supplement: Supplementary file 1 [file ijms-24-06788-s001.zip › ijms-2255803-supplementary.pdf]

Table of Contents of Supplementary Figures

Figure S1 1

Figure S2 2

Figure S3 3

Figure S4 4

Figure S5 5

Figure S5 6

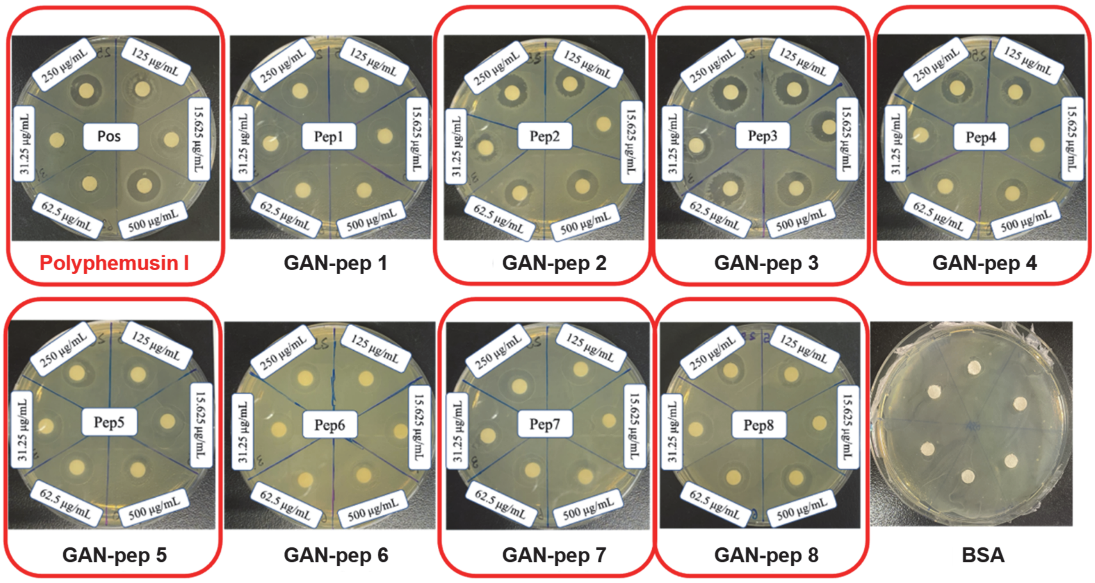

Figure S1. Growth inhibition test against *E. coli* with peptides at different concentrations. Peptides are highlighted with red rectangles if inhibition zones occur around the disks. Here, polyphemusin I is a known AMP with broad-spectrum activity against various microorganisms as a positive control.

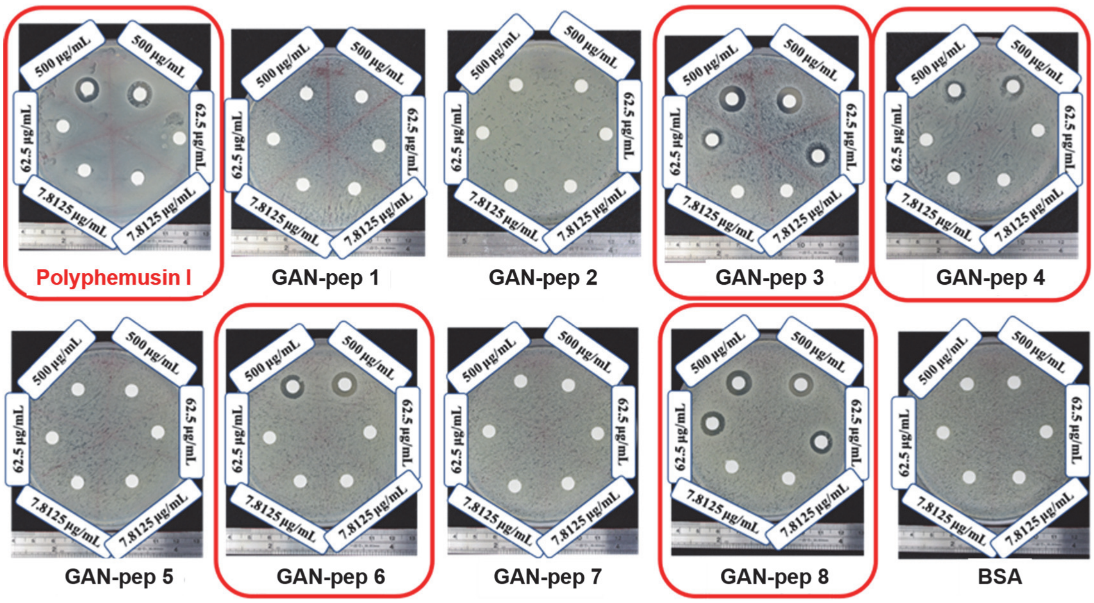

Figure S2. Growth inhibition test against methicillin-susceptible *S. aureus* (MSSA) with peptides at different concentrations. Peptides are highlighted with red rectangles if inhibition zones occur around the disks. Here, polyphemusin I is a known AMP with broad-spectrum activity against various microorganisms as a positive control.

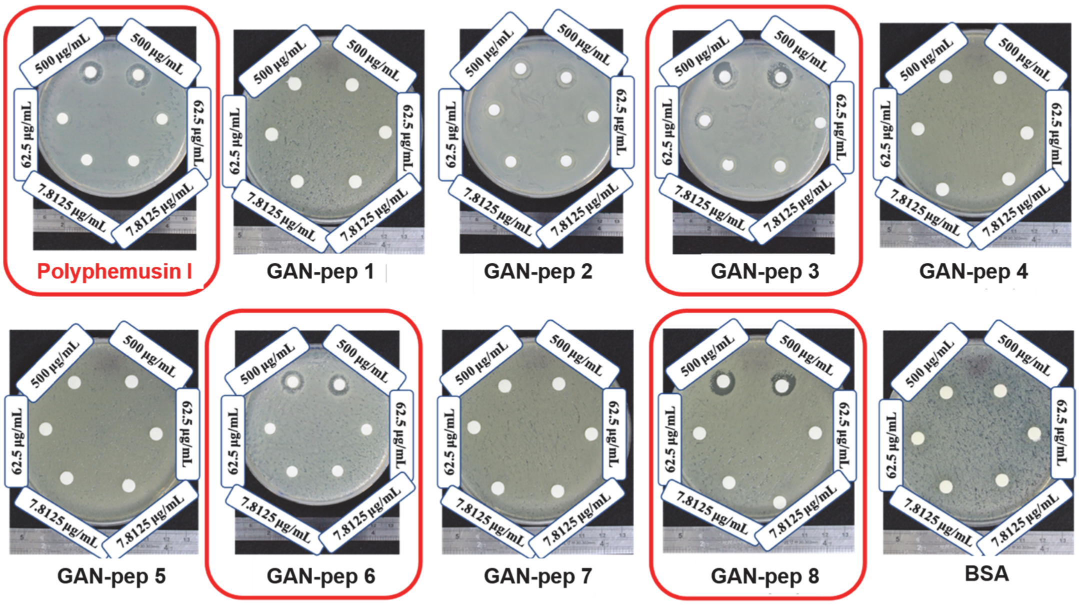

Figure S3. Growth inhibition test against methicillin-resistant *S. aureus* (MRSA) with peptides at different concentrations. Peptides are highlighted with red rectangles if inhibition zones occur around the disks. Here, polyphemusin I is a known AMP with broad-spectrum activity against various microorganisms as a positive control.

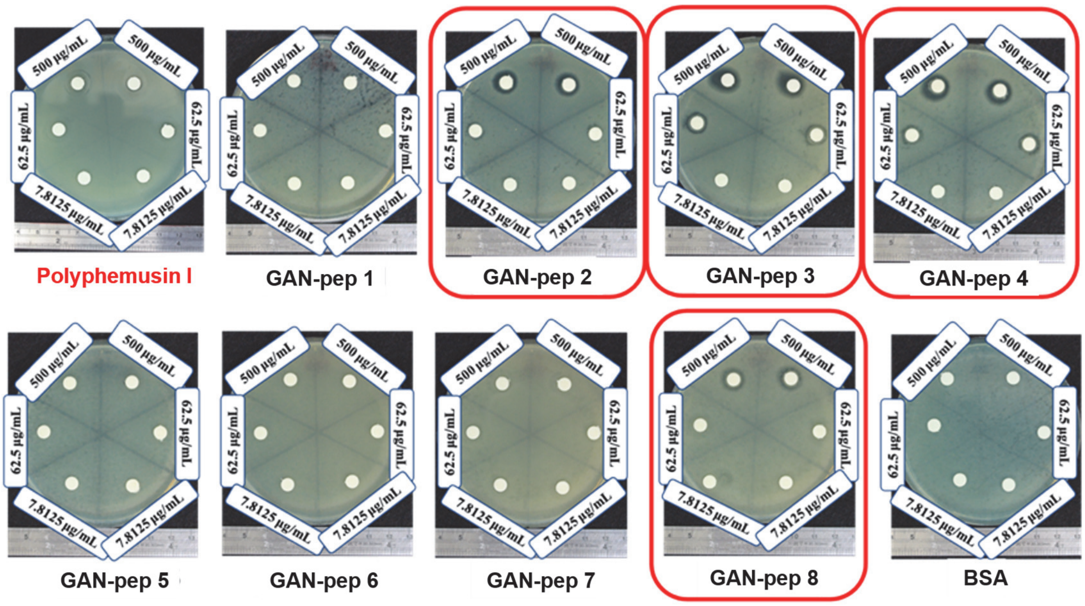

Figure S4. Growth inhibition test against carbapenem-susceptible *P. aeruginosa* with peptides at different concentrations. Peptides are highlighted with red rectangles if inhibition zones occur around the disks. Here, polyphemusin I is a known AMP with broad-spectrum activity against various microorganisms.

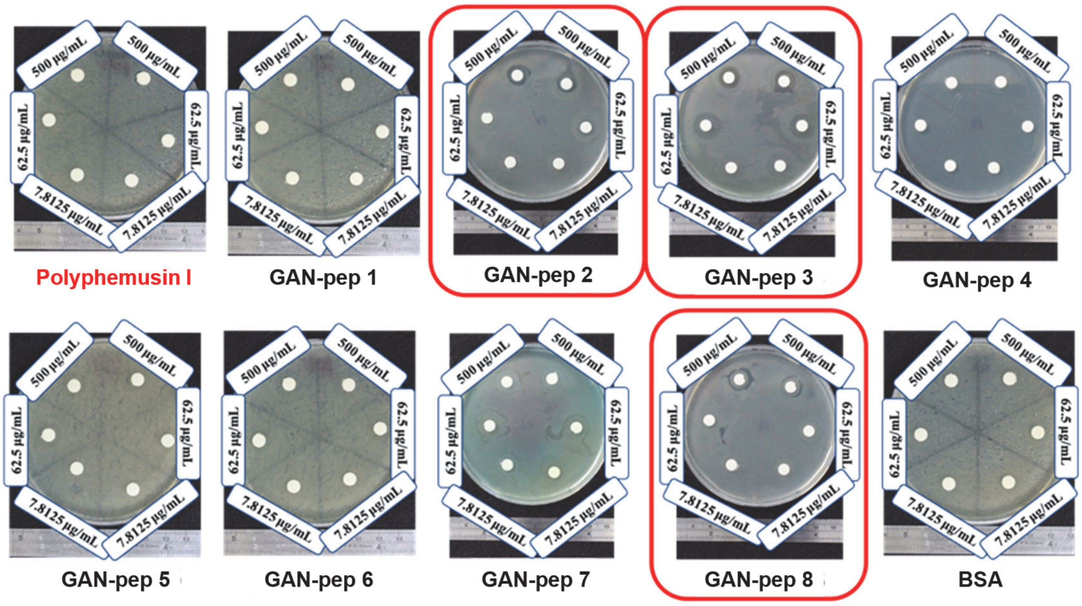

Figure S5. Growth inhibition test against carbapenem-resistant *P. aeruginosa* with peptides at different concentrations. Peptides are highlighted with red rectangles if inhibition zones occur around the disks. Here, polyphemusin I is a known AMP with broad-spectrum activity against various microorganisms.

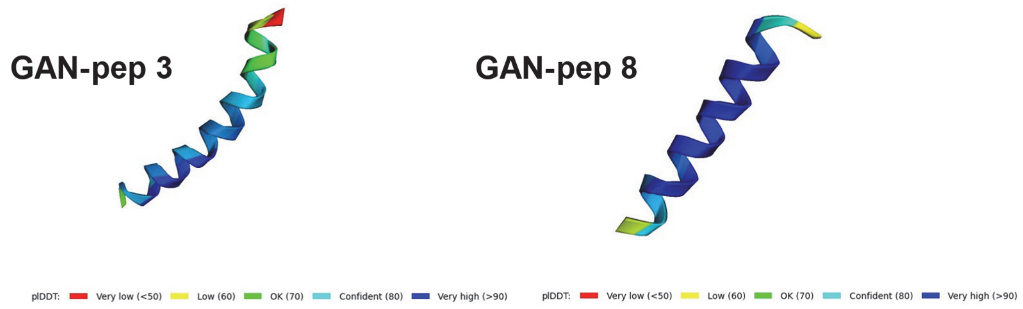

Figure S6. The structure prediction in aplhafold2 and GAN-pep 3 and 8 show the helix structures. pLDDT stands for predicted local distance difference test score in Alphafold2. It is a measure used to evaluate the accuracy and reliability of protein structure predictions generated by deep learning models. pLDDT scores range from 0 to 100, with higher scores represented in cyan and blue, indicating better model accuracy.
